# The link between diabetic retinal and renal microvasculopathy is associated with dyslipidemia and upregulated circulating level of cytokines

**DOI:** 10.3389/fpubh.2022.1040319

**Published:** 2023-01-17

**Authors:** Xiaosi Chen, Xinyuan Zhang, Zhizhong Gong, Yang Yang, Xiaohong Zhang, Qiyun Wang, Yanhong Wang, Rui Xie

**Affiliations:** ^1^Beijing Tongren Eye Center, Beijing Tongren Hospital, Capital Medical University, Beijing, China; ^2^Beijing Retinal and Choroidal Vascular Diseases Study Group, Beijing, China; ^3^Division of Medical Affairs, Beijing Hospital of Traditional Chinese Medicine, Capital Medical University, Beijing, China; ^4^Clinical Laboratory of Beijing Tongren Hospital, Capital Medical University, Beijing, China; ^5^Department of Epidemiology and Biostatistics, School of Basic Medicine, Peking Union Medical College, Institute of Basic Medical Sciences, Chinese Academy of Medical Sciences, Beijing, China

**Keywords:** dyslipidemia, diabetes mellitus, diabetic retinopathy, fasting blood glucose, cytokines, diabetic kidney disease, Albumin-to-creatinine ratio

## Abstract

**Purpose:**

To investigate the mechanisms underlying the correlations between diabetic retinopathy (DR) and diabetic nephropathy (DKD) and examine whether circulating cytokines and dyslipidemia contribute to both DR and DKD in patients with 2 diabetes mellitus (T2DM).

**Methods:**

A total of 122 patients with T2DM were enrolled and categorized into the DM group (without no DR and DKD), DR group [non-proliferative DR (NPDR), and proliferative DR (PDR)] with no DKD), DR complicated with DKD groups (DR+DKD group). The biochemical profile, including fasting blood glucose (FBG), glycated hemoglobin (HbA1c), and lipid profile were estimated, and plasma inflammatory and angiogenic cytokines [monocyte chemoattractant protein-1 (MCP-1), interleukin (IL)-6, IL-8, vascular endothelial growth factor (VEGF)-A, C, D, and placental growth factor (PlGF)] were analyzed by protein microarrays. The atherogenic plasma index (API) was defined as low-density lipoprotein cholesterol (LDL-C)/high-density lipoprotein-cholesterol (HDL-C); atherogenic index (AI) was calculated as [(total cholesterol (TC) -HDL-C)/HDL-C], and atherogenic index of plasma (AIP) was defined as log (TG/HDL-C).

**Results:**

By multivariable disordered regression analysis, after controlling for duration of DM and hypertension, LDL-C (*p* = 0.019) and VEGF-D (*p* = 0.029) resulted as independent risk factors for DR. Albumin-to-creatinine ratio (uACR) (*p* = 0.003) was an independent risk factor for DR with DKD. In DR, NPDR, and PDR groups, grades of A1, A2, and A3 of albuminuria increased with the severity of DR. In A1, A2, and A3 grade groups, the severity of DR (DM, NPDR, and PDR) increased with higher albuminuria grades. Kendall's tau-b correlation coefficient analysis revealed that FBG (*p* = 0.019), circulating level of PlGF (*p* = 0.002), and VEGF-D (*p* = 0.008) were significantly positively correlated with the grades of uACR (*p* < 0.001), and uACR grades were significantly correlated with DR severity (*p* < 0.001).

**Conclusions:**

The occurrence and severity of DR are closely correlated with kidney dysfunction. Among the three kidney functional parameters, uACR resulted as the better indicator of DR severity and progression than glomerular filtration (eGFR) and serum creatinine (Scr). Impaired FBG was associated with microalbuminuria, emphasizing that well-controlled FBG is important for both DR and DKD. The link between diabetic retinal and renal microvasculopathy was associated with dyslipidemia and upregulated circulating level of angiogenic cytokines.

## 1. Introduction

Diabetes Mellitus (DM) is the most common noncommunicable epidemiological illness and a major public health problem that has been approaching epidemic proportions globally. China ranks number one, with the highest number of people with DM (1). The high mortality and disability rates caused by the various complications of diabetes impose a heavy economic burden on society. Thus, early diagnosis and time prevention of complication is of extreme importance.

Diabetic retinopathy (DR), diabetic kidney disease (DKD), and diabetic peripheral neuropathy are the most prevalent microvascular complications of type 2 diabetes (T2DM). DR and DKD have complicated interleaving relationships and are the main causes of death and disability in diabetic patients. In addition, DR is a leading cause of blindness in the working-age population (2). The META-EYE Study Group reported that the prevalence of DR is 34.6% worldwide, while the prevalence of vision-threatening proliferative DR that can lead to blindness is 10.2%, accounting for 51% of blindness cases worldwide (3).

DKD has an insidious onset and lacks distinctive clinical signs, and it is responsible for 20–40% of cases of DM (4–7). Its timeline is well characterized for type 1 diabetes mellitus (i.e., DKD develops within 10 years of the first onset of type 1 DM); in those with T2DM, it usually starts developing after the onset of hyperglycemia. Furthermore, DKD remains a leading cause of new-onset end-stage renal disease of DM (accounting for 50.1%) and is the leading cause of mortality (6, 8). Currently, there are still limited treatments for DR and DKD.

Numerous international large-scale epidemiological studies, including the UK Prospective Diabetes Study (UKPDS, follow up for 10 years) (9), The Action in Diabetes and Vascular Disease: Preterax and Diamicron Modified Release Controlled Evaluation (ADVANCE, follow up for 4.3 years) (10), the Action to Control Cardiovascular Risk in Diabetes (ACCORD, follow up for 4 years) (11), and the Veteran Affairs Diabetes Trial (VADT, follow up for 5 years) (12) have demonstrated that even though the multiple risk factor interventions (hyperglycemia, blood pressure, and lipid regulation) can effectively reduce the risk of diabetic microvascular disease (DMVC), 51% of diabetic patients still develop DR (51% of those with DR) and DKD (25% of individuals with DKD) (13). DMVC (the residual risk of DMVC) that still exists after comprehensive management of diabetic patients is a major challenge for both ophthalmologists and endocrinologists.

The interrelationships between DR and DKD are currently receiving extensive attention. Several studies have suggested a close connection between the occurrence and progression of the two common diabetic microvasculopathies. According to a large-scale epidemiological investigation conducted by the Chinese Center for Disease Control and Prevention (CDC), the number of those suffering from both DR and DKD might exceed 2.5 million (14). According to the findings of a national cross-sectional study conducted by Jia's team in 2016, the prevalence of high level of proteinuria in patients with DR reached 47.8% among the 3,301 patients with T2DM (average age 59.34 ± 12.28 years, average DM course of 8.48 years) and the frequency of DR increased as urine albumin levels increased (15). The Wisconsin Epidemiologic Study of Diabetic Retinopathy (WESDR) study also found that microalbuminuria was statistically significantly associated with proliferative diabetic retinopathy (PDR) and clinically significant macular in the younger group by multivariable analysis (4). Two prospective studies on the Singaporean population (*N* = 5,763, age ≥ 40 years) found that retinal microvasculopathy was associated with the risk of end-stage renal illness, and end-stage renal disease was 2.6 times more likely to occur in person with DR than in patients without retinopathy (16).

Although the complicated interrelationships between DR and DKD have been elucidated in numerous studies, the underlying mechanisms remain still uncertain. Our previous study suggested that homocysteine contributes to DR and is associated with increased urine microalbumin (17). In this study, we further investigated the correlations between DR and DKD in patients with T2DM, testing the hypothesis that known circulating angiogenic and inflammatory cytokines and dyslipidemia contribute to both DR and DKD.

## 2. Materials and methods

### 2.1. Participants

This prospective study followed the principles of the Declaration of Helsinki and was approved by the Ethics Committee of Beijing Tongren Hospital, Capital Medical University. All subjects signed an informed consent form before participation.

A total of 122 participants with T2DM, including 73 males and 49 females, aged 24–76 years old, were recruited from the outpatient department of Beijing Tongren Hospital from April 2016 to September 2020. Age, gender and duration of diabetes, and other related data were also collected.

### 2.2. Inclusion and exclusion criteria

Inclusion were the following: (1) patients diagnosed with T2DM and DR according to the 2016 American Diabetes Association (ADA) guidelines of DM (18) and 2002 A Position Statement of DR (19); those who were able to provide informed consent. Exclusion criteria were: those with T2DM with macular edema secondary to other retinal vascular diseases; co-existent other retinal diseases such as age-related macular degeneration, uveitis, and inherited retinal diseases; recent history of posterior segment or cataract surgery; ocular media opacity and unable to tolerate examinations due to severe system diseases. Also, T2DM with normal fundus but with abnormal estimated glomerular filtration (eGFR) or Albumin-to-creatinine ratio (uACR) were not considered. Patients with a history of other chronic kidney diseases were also excluded.

### 2.3. Extensive eye examinations

Best-corrected visual acuity (BCVA) and non-contact intraocular pressure (TX20 Automatic Non-contact Tonometer, Canon Co., Ltd, Tokyo, Japan) assessment, slit-lamp microscopic examination (SL-IE Slit Lamp Microscope, Topcon Co., Ltd, Tokyo, Japan), and fundus examination with mydriasis were applied for all the participants. Fundus photography (CR-1 non-mydriatic Fundus Camera, Canon Co., Ltd) was used to capture at least two fields centered on both eyes' optic disc and macula. Two independent ophthalmologists (Q.W. with 4 and B.Q. with 6 years of experience in the field) ascertained the DR status of the participants based on the International DR severity scale (19). Swept-source optical coherent tomography was applied (DRI OCT1 Atlantis scanner, Topcon Co., Ltd., Tokyo, Japan or Plex Elite 9000, Carl Zeiss Meditec, Inc, Oberkochen, German) for all the enrolled subjects. B-scan images were obtained by a 9 mm × 9 mm scanning range mode. The DR status of the worse eye was recorded as an individual's DR grade.

### 2.4. Definition and classification of diabetic kidney disease

DKD was defined as “a clinical diagnosis made based on the presence of albuminuria and/or reduced eGFR in the absence of signs or symptoms of other primary causes of kidney damage” according to the ADA “Standards of Medical Care in Diabetes 2022.” uACR was used to evaluate the severity of albuminuria; high urinary albumin excretion was defined as ≥30 mg/g Cr. As recommended by ADA, eGFR was calculated by the Chronic Kidney Disease Epidemiology Collaboration (CKD-EPI) equation (20); persistent >60 ml/min/1.73 m^2^ was defined as normal (20).

Stages of DKD were graded according to the Kidney Disease: Improving Global Outcomes (KDIGO) classification criteria with incorporates albuminuria at all stages of eGFR. In this system, DKD is classified based on the cause (C), GFR (G), and albuminuria (A). GFR categories included (G1-G5): G1: normal to high (GFR≥90 ml/min/1.73 m^2^), G2: mildly decreased 60–89 ml/min/1.73 m^2^), G3: mildly to moderately decreased (45–59 ml/min/1.73 m^2^), G4: moderately to severely decreased (30–44 ml/min/1.73 m^2^), G5: severely decreased (15–29 ml/min/1.73m^2^) and kidney failure (< 15 ml/min/1.73 m^2^); albuminuria categories included three classes (A1–A3): A1: normal to mildly (< 30 mg/g), A2: moderately (30–299 mg/g) and A3: severely increased (≥300 mg/g) (20).

### 2.5. Determination of biochemistry profile and plasma cytokines

Blood biochemistry profile: fasting biochemical examination was performed for all the participants. These included low-density lipoprotein cholesterol (LDL-C), high-density lipoprotein-cholesterol (HDL-C), triglycerides (TG), total cholesterol (TC), glycated hemoglobin (HbA1c), fasting blood glucose (FBG) etc. Atherogenic index (AI) was defined as (TC-HDL-C)/HDL-C; atherogenic plasma index (API) was calculated as LDL-C/HDL-C; atherogenic index of plasma (AIP) was calculated as log (TG/HDL-C).

To determine the plasma level of angiogenic and inflammatory cytokines, including vascular endothelial growth factor (VEGF)-A, VEGF-C, VEGF-D, placental growth factor (PlGF), monocyte chemoattractant protein-1 (MCP-1), interleukin (IL)-6 and IL-8, the Luminex technology (Luminex 200™ liquid chip detector, Millipore, Boston, Massachusetts, USA) was applied according to the manufacturer's instructions.

### 2.6. Subgrouping of the participants

According to the 2020 ADA guidelines of DM (18) and 2017 A Position Statement of DR (13), the participants were assigned to the DM group {no DR, 32 patients, aged 37–75 years, median [interquartile range (IQR)]: 56 (48–65) years}, non-proliferative diabetic retinopathy group [NPDR group) [56 patients, aged 29–76 years, 56 (51–61)] years], and PDR group [43 patients, aged 27–74 years, 55 (49–60) years].

According to the definition of DR and DKD, all participants were further grouped to DM (no DR and DKD), DR (no DKD), DKD (no DR), and DR+DKD groups. The DR participants were further categorized into “PDR” group if they had retinal and/or optic disc neovascularization in at least one eye. Those with any other DR grade were categorized as the NPDR group, and participants with no DR in both eyes were assigned to the “DM” group. DR and DKD were classified according to the criteria according to the 2020 ADA guidelines of DM (18) and 2017 ADA: A Position Statement of DR (21) as described above.

### 2.7. Determination of the cutoff value of AIP, API, and AI by receiver operating characteristic (ROC) curve

API (LDL-C/HDL-C), AI [(TC-HDL-C)/HDL-C), and AIP (log(TG/HDL-C)] with high sensitivity and specificity at maximum Youden index were selected as the cutoff values on the ROC curve as described previously. Patients with API > 2.24 (AUC: 0.746; sensitivity = 0.708, specificity = 0.517), AI > 2.91 (AUC, 0.723; sensitivity = 0.629; specificity = 0.724) or AIP > 0.01 (AUC 0.564; sensitivity = 0.607, specificity = 0.552) were assigned to high API, high AI, and high AIP groups, respectively.

### 2.8. Sample size calculation

Power Analysis and Sample Size software (PASS 2022, NCSS LLC, Utah, USA) were used to determine the sample size as we previously described (17). The sample size was calculated at a 95% confidence level with a margin of error of +/− 5% and designed power (1-beta = 85%, the actual power was 86.59%). Based on our pilot study, as the representing parameter of the study group, the mean level of LDL-C was 3.17 and 2.46 in the control group (DM), respectively; the mean, the standard deviation was +−0.75, the minimum sample per arm (per group) was 22 subjects.

### 2.9. Statistical analysis

SPSS software (SPSS, Inc. 23.0, Chicago, IL, USA) was applied for statistical analysis. Kolmogorov-Smirnov test and Shapiro-Wilk test were used to assess data normality. Variance homogeneity was tested by Levene's test. Age of participants, duration of diabetes, and biochemical parameters were described as means ± standard deviation (mean ± SD) or median (IQR). One-way analysis of variance (ANOVA) or Kruskal-Wallis test were used for group comparisons. The circulating levels of cytokines VEGF-A, VEGF-C, VEGF-D, PlGF, MCP-1, IL-6, and IL-8 were described as mean ± SD or median (IQR); group comparisons (DM, NPDR, and PDR groups, or DM, DR, DR+DKD groups) were analyzed by independent sample *t*-test or Mann–Whitney *U*-test according to the data distribution. Bonferroni corrections were applied for comparison between the groups. The Kendall's Tau-b rank correlation coefficient was used for testing the correlations between the cytokines or chemical parameters, classification of DKD, and different grades of DR. Single ordinal logistic regression analysis was applied to assess the influence of the variables on DR or DKD. Multivariable ordinal logistic regression was applied to evaluate the effects of the variables on DR or uACR grading. Multivariable logistic regression analysis was used to evaluate the effects of the cytokines on different groups. A *p* < 0.05 indicated statistical significance.

## 3. Results

### 3.1. Baseline demographic and biochemical profile characteristics

A total of 122 participants with T2DM were included in the study. The participants were assigned to the DM group if they had no DR or DKD (23 patients, aged 24–76 years, median (IQR): 57 (53–65) years), DR group if they had DR but no DKD [35 patients, aged 27–71 years, 56 (49–60) years], and DR+DKD group if they had both DR and DKD [64 patients, aged 29–76 years, 55 (49–62.75) years]. 9 T2DM patients [aged 40–76 years, 49 (42–70) years] with normal fundus but with abnormal eGFR or uACR(the DKD group) were not considered in this study, because these patients cannot be excluded from other primary causes of kidney damage unless confirmed by a kidney biopsy, which was not accepted by those patients. Significant differences in the duration of DM (*p* = 0.041), LDL-C (*p* = 0.022), API (*p* = 0.022), FBG (*p* = 0.031), Scr (*p* = 0.027), eGFR (*p* = 0.025) and uACR (*p* < 0.001) were found among the three groups. There was no significant difference in age (*p* = 0.453), gender (*p* = 0.720) and duration of hypertension (*p* = 0.955) among the three groups (Table 1).

**Table 1:** Comparison of baseline demographic and clinical characteristics in subjects with DM, DR, and DR+DKD.

	**DM**	**DR**	**DR+DKD**	**H/χ2/F**	***p***
Number	23	35	64	/	*/*
Age, years	57.00 (53.00–65.00)	56.00 (49.00–60.00)	55.00 (49.00–62.75)	1.58^b^	0.453
Gender (Male/Female)	14/9	19/16	40/24	0.65^c^	0.720
Duration of DM, years	10.00 (2.00–15.00)	12.00 (10.00–16.00)	12.50 (8.00–19.50)	6.37^b^	0.041^*^
Duration of HBP, years	0 (0–5.00)	1.00 (0–7.00)	1.00 (0–7.00)	0.09^b^	0.955
TC, mmol/L	4.31 ± 0.76	5.03 ± 1.23	4.84 ± 1.20	2.86^a^	0.061
TG, mmol/L	1.42 ± 1.08	1.73 ± 1.20	1.52 ± 0.97	0.67^a^	0.513
LDL-C, mmol/L	2.45 (1.91–3.07)	3.15 (2.47–3.70)	2.88 (2.16–3.72)	7.63^b^	0.022^*^
HDL-C, mmol/L	1.24 (1.02–1.43)	1.13 (0.98–1.48)	1.15 (0.98–1.46)	0.38^b^	0.260
AI (TC-HDL-C)/HDL-C)	2.53 (1.80–3.11)	3.04 (2.20–4.27)	2.96 (2.44–3.67)	5.30^b^	0.071
API (LDL-C/HDL-C)	2.17 (1.54–2.42)	2.70 (1.97–3.33)	2.47 (1.89–3.03)	7.61^b^	0.022^*^
AIP (log (TG/HDL-C)	−0.02 (−0.27–0.07)	−0.01 (−0.22–0.39)	0.03 (−0.20–0.23)	1.32^b^	0.180
HbA1c, %	6.60 (6.10–8.90)	7.70 (7.00–8.90)	7.80 (6.70–9.08)	4.08^b^	0.130
FBG, mmol/L	6.48 (5.59–8.18)	8.10 (6.37–9.45)	8.34 (6.53–11.29)	6.95^b^	0.031^*^
Scr, μmol/L	66.00 (51.20–80.80)	59.90 (47.00–69.60)	71.45 (54.63–91.08)	7.21^b^	0.027^*^
eGFR, mL/min	103.85 (91.56–124.79)	123.79 (96.92–155.60)	101.47 (75.11–138.97)	7.41^b^	0.025^*^
uACR, mg/g Cr	13.14 (4.62–19.56)	10.31 (7.08–18.44)	214.99 (44.12–1173.91)	85.61^b^	< 0.001^*^

^*^Statistically significant: p < 0.05. According to the type of data and the data distribution,

^a^one-way ANOVA analysis,

^b^Kruskal-Wallis analysis,

^c^Chi-square test were applied. DM, Diabetes mellitus; DR, Diabetic retinopathy; DKD, Diabetic Kidney Disease; HBP, High blood pressure; TC, total cholesterol; TG, triglycerides; HDL-C, High-density lipoprotein Cholesterol; LDL-C, Low-density lipoprotein Cholesterol; AI, Atherosclerosis index [(TC-HDL-C)/HDL-C]; AIP, Atherogenic index of plasma [log (TG/HDL-C)]; API, Atherogenic plasma index (LDL-C/HDL-C); HbA1c, Hemoglobin; FBG, fasting blood glucose; Scr, Serum creatinine; eGFR, Estimated Glomerular Filtration Rate; uACR, Urinary albumin/creatinine ratio.

**Figure 1:**
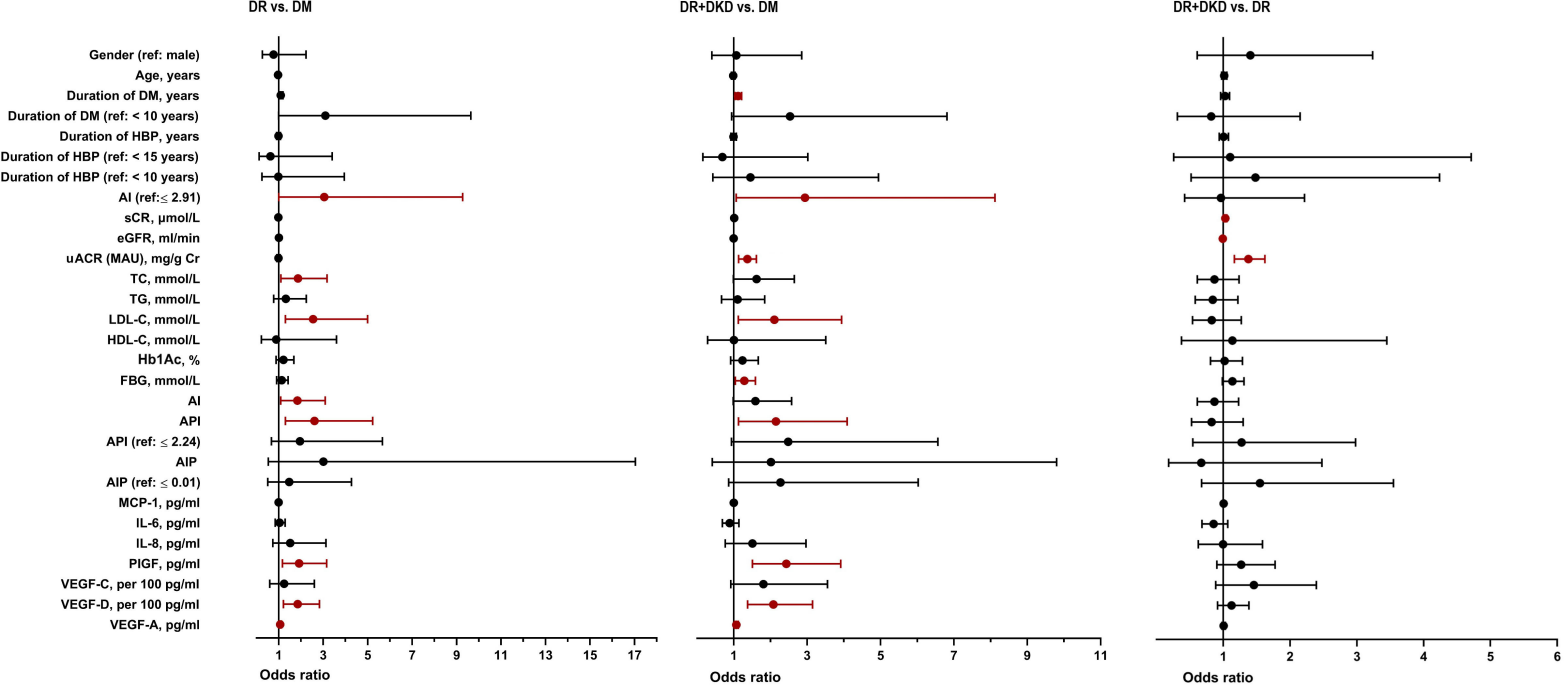
Univariate logistic regression analysis between DM, DR, and DR+DKD groups. DM, diabetes mellitus; DR, diabetic retinopathy; DKD, diabetic kidney disease; HBP, high blood pressure; TC, total cholesterol; TG, triglycerides; HDL-C, high density lipoprotein cholesterol; LDL-C, low-density lipoprotein cholesterol; AI, atherosclerosis index [(TC-HDL-C)/HDL-C]; AIP, atherogenic index of plasma [log (TG/HDL-C)]; API, atherogenic plasma index (LDL-C/HDL-C); HbA1c, hemoglobin; FBG, fasting blood glucose; Scr, serum creatinine; eGFR, estimated glomerular filtration rate; uACR, urinary albumin/creatinine ratio; MCP-1, monocyte chemoattractant protein-1; IL-6, interleukin-6; IL-8, interleukin-8; PlGF, placental growth factor; VEGF, vascular endothelial growth.

By multinomial logistic regression analysis, when DM was considered as the reference, there was a significant difference in AI (*p* = 0.022), AI grouping (when it is higher than 2.91, *p* = 0.050), TC (*p* = 0.021), LDL-C (*p* = 0.007), and API (*p* = 0.007) between the DR and DM groups. There was no significance in gender (*p* = 0.621), age (*p* = 0.269), duration of DM (*p* = 0.055) and hypertension (*p* = 0.778), TG (*p* = 0.312), HDL-C (*p* = 0.862), Hb1Ac (*p* = 0.244), FBG (*p* = 0.271) between the DR and DM groups. There was a significant difference in DM duration (*p* = 0.008), AI grouping (when it is higher than 2.91, *p* = 0.038), uACR (OR = 1.355, 95% CI 1.147–1.600, *p* < 0.001), LDL-C (*p* = 0.020), FBG (*p* = 0.019) and API (*p* = 0.020) between the DR+DKD and DM groups. uACR (OR = 1.375, 95% CI 1.165–1.623, *p* < 0.001), Scr (OR = 1.030, 95% CI 1.008–1.051, *p* = 0.007) and eGFR (OR = 0.991, 95% CI 0.983–0.998, *p* = 0.017) was significantly higher in DR+DKD group in comparison with DR group (Figure 1).

**Figure 2:**
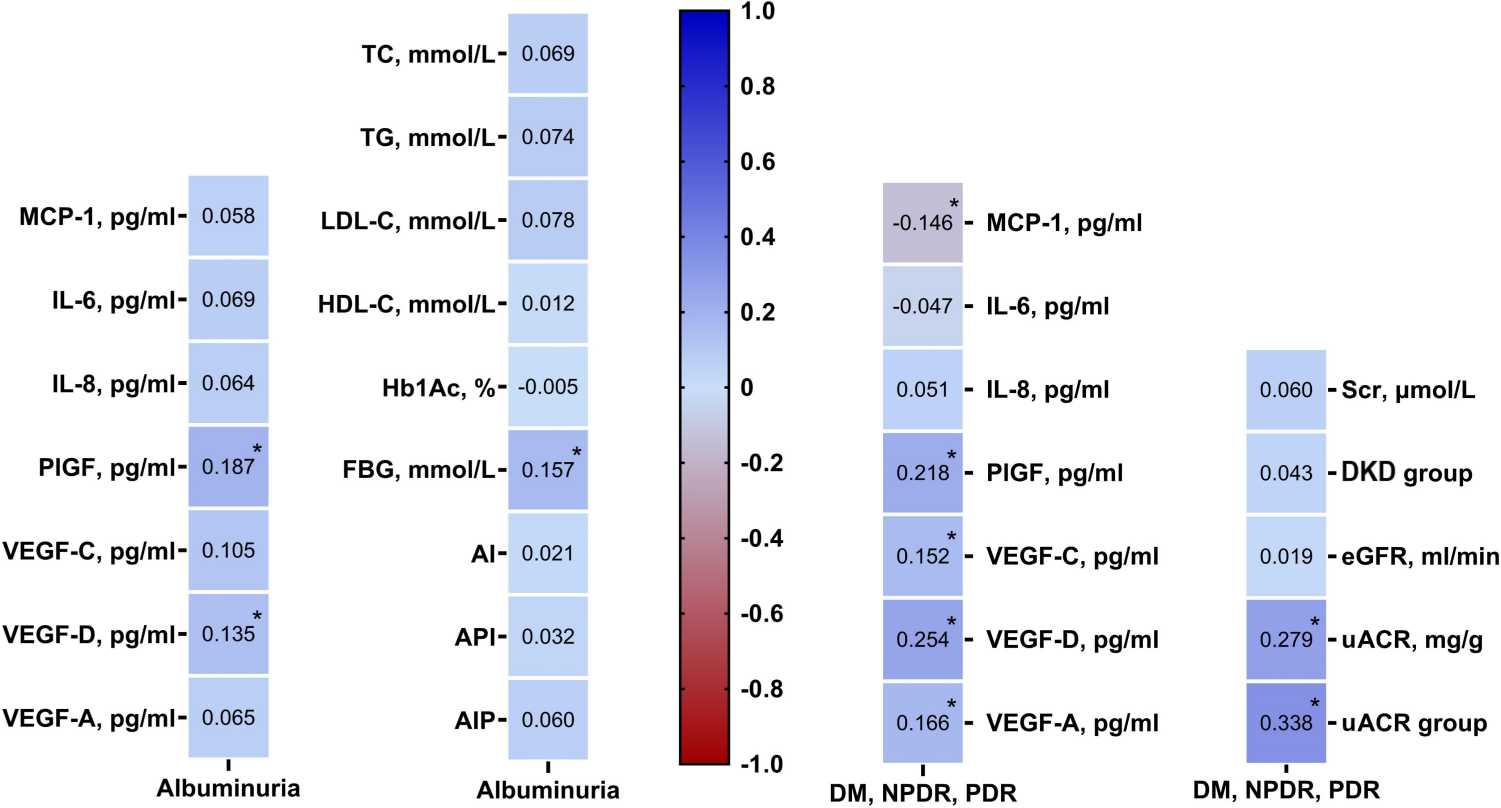
Kendall's Tau-b rank correlation coefficient analysis showing angiogenic cytokines were associated with DR and DKD grades, renal function profiles were related with DR grades. DM, diabetes mellitus; DR, diabetic retinopathy; DKD, diabetic kidney disease; TC, total cholesterol; TG, triglycerides; HDL-C, high density lipoprotein cholesterol; LDL-C, low-density lipoprotein cholesterol; AI, atherosclerosis index [(TC-HDL-C)/HDL-C]; AIP, atherogenic index of plasma [log (TG/HDL-C)]; API, atherogenic plasma index (LDL-C/HDL-C); HbA1c, hemoglobin; FBG, fasting blood glucose; Scr, serum creatinine; eGFR, estimated glomerular filtration rate; uACR, urinary albumin/creatinine ratio; MCP-1, monocyte chemoattractant protein-1; IL-6, interleukin-6; IL-8, interleukin-8; PlGF, placental growth factor; VEGF, vascular endothelial growth.

According to Kendall's tau-b correlation coefficient analysis, only FBG was significantly positively correlated with the grades of uACR (r = 0.157, *p* = 0.019). Other parameters including TC (*p* = 0.339), TG (*p* = 0.253), LDL-C (*p* = 0.268), HDL-C (*p* = 0.339), Hb1Ac (*p* = 0.942), AI (*p* = 0.756), API (*p* = 0.630), AIP (*p* = 0.367) were positively correlated with the grades of uACR, but there was no statistically difference (Figure 2).

### 3.2. Associations of a renal function profile among the DM, DR, and DR+DKD groups

To test the hypothesis that renal impairment is associated with the pathogenesis of DR and DKD, three renal function parameters were compared between the three groups by the Kruskal-Wallis H test in this study. There was significant differences in the most clinical used renal functional parameters serum creatinine (Scr, *p*_*all*_ = 0.027, *p*_*DMvs*.*DR*+*DKD*_ = 0.023), glomerular filtration (eGFR, *p*_*all*_ = 0.025, *p*_*DRvs*.*DR*+*DKD*_ = 0.020) and urine microalbumin-creatinine ratio (uACR, *p*_*all*_ < 0.001, *p*_*DM*_ _*vs*.*DR*_ < 0.001, *p*_*DM*_ _*vs*.*DR*+*DKD*_ < 0.001) between the DM, DR and DR+DKD groups, indicating that uACR is more sensitive and closer renal dysfunctional parameters in the diabetic microvascular complications especially for patients with DR and DKD (Table 1).

When DR was considered as the reference, multinomial logistic regression analysis showed a significant difference in the three renal function parameters Scr (*p* = 0.007), eGFR (*p* = 0.017) and uACR (*p* < 0.001) between the DR+DKD and DR groups. There was no statistical significance in the lipid profile and other baseline parameters between the two groups (Figure 1).

### 3.3. The correlations between a renal profile with DR severity

To further investigate if uACR is associated with DR severity, the same cohort was categorized into DM (without DR), NPDR, and PDR groups according to the criteria described above. By using Kendall's tau-b/c (Tb or Tc) correlation coefficient analysis, among all the indicators/parameters of renal function (Scr, eGFR, uACR, and grades of uACR), uACR (r = 0.279, *p* < 0.001) and uACR grades (r = 0.338, *p* < 0.001) were significantly correlated with DR severity. This result indicates that uACR is closely correlated with the DR severity and is a good indicator of DR progression (Figure 2).

### 3.4. Associations of plasma inflammatory and angiogenic cytokines with DM, DR, DR+DKD groups

We further investigated the effects of inflammatory and angiogenic cytokines on both DR and DKD from a global perspective. Interestingly, there was no statistical difference in the inflammatory cytokines between the DM, DR, and DR+DKD groups, but PlGF (*p*_all_ < 0.001, *p*_*DMvs*.*DR*_ < 0.001*, p*_*DMvs*.*DR*+*DKD*_ = 0.031, *p*_*DRvs*.*DR*+*DKD*_ = 0.031), VEGF-D (*p*_*all*_ = 0.001, *p*_*DRvs*.*DM*_ = 0.012, *p*_*DR*+*DKDvs*.*DM*_ < 0.001) and VEGF-A (*p*_*all*_ = 0.002, *p*_*DRvs*.*DM*_ = 0.006, *p*_*DR*+*DKDvs*.*DM*_ = 0.002) were significantly different among the three groups. The results indicated that angiogenic cytokines, not inflammatory cytokines, are differently regulated and contribute to diabetic microvasculopathy (Table 2).

**Table 2:** Comparison of plasma inflammatory and angiogenic cytokines in subjects with DM, DR, and DR+DKD.

	**DM**	**DR**	**DR+DKD**	**H/χ2/F**	***p***
MCP-1, pg/ml	250.59 (240.48–295.80)	247.61 (227.50–270.13)	249.94 (227.91–287.57)	1.23^b^	0.540
IL-6, pg/ml	0.70 (0.28–1.22)	0.55 (0.24–1.74)	0.63 (0.29–1.55)	0.19^b^	0.908
IL-8, pg/ml	0.91 (0.61–1.72)	1.37(1.02–1.99)	1.27 (0.98–1.65)	3.71^b^	0.157
PlGF, pg/ml	1.55 ± 1.27	2.40 ± 1.40	2.77 ± 1.10	8.47^a^	< 0.001^*^
	*p_*DMvs*.*DR*_* < 0.001; *p_*DMvs*.*DR*+*DKD*_ =* 0.031; *p_*DRvs*.*DR*+*DKD*_ =* 0.031				
VEGF-C, pg/ml	51.41 (8.58–148.15)	93.20 (55.69–122.65)	98.14 (53.81–182.03)	4.36^b^	0.113
VEGF-D, pg/ml	135.96 (14.73–248.50)	252.01 (131.06–395.90)	262.85 (164.43–435.87)	14.79^b^	0.001*
	*p_*DMvs*.*DR*_* = 0.012; *p_*DMvs*.*DR*+*DKD*_* < 0.001				
VEGF-A, pg/ml	15.22 (9.62–26.27)	27.64 (19.99–32.91)	25.23 (19.65–35.98)	12.55^b^	0.002^*^
	*p_*DMvs*.*DR*_ =* 0.002; *p_*DMvs*.*DR*+*DKD*_* = 0.006				

^*^Statistically significant: p < 0.05. According to the type of data and the data distribution,

^a^one-way ANOVA analysis,

^b^Kruskal-Wallis analysis. DM, Diabetes mellitus; DR, Diabetic retinopathy; DKD, Diabetic Kidney Disease; MCP-1, Monocyte chemoattractant protein-1; IL-6, Interleukin- 6; IL-8, Interleukin- 8; PlGF, Placental growth factor; VEGF, Vascular endothelial growth.

When DM was considered as the reference, multinomial logistic regression analysis indicated that PlGF (*p* = 0.010), VEGF-D per 100 (*p* = 0.004) and VEGF-A (*p* = 0.021) were significantly different between the DR and DM groups; yet, there was no significant difference in the inflammatory cytokines between the two groups (Figure 1). When DM was considered as the reference, multinomial logistic regression analysis indicated that PlGF (*p* < 0.001), VEGF-D per 100 (*p* = 0.001) and VEGF-A (*p* = 0.010) were significantly different between the DR+DKD and DM groups. Also, there was no significant difference in the inflammatory or angiogenic cytokines between the DR+DKD and DR groups (Figure 1).

By Kendall's tau-b correlation coefficient analysis, PlGF (r = 0.187, *p* = 0.005) and VEGF-D (r = 0.135, *p* = 0.048) were significantly positively correlated with the grades of uACR. Other circulating cytokines, including MCP-1 (*p* = 0.432), IL-6 (*p* = 0.313), IL-8 (*p* = 0.361), VEGF-C (*p* = 0.120), and VEGF-A (r = 0.07, *p* = 0.345) were found to be positively correlated with DKD, but there was no statistical significance (Figure 2).

### 3.5. Associations of plasma inflammatory and angiogenic cytokines with DR severity

Kendall's tau-b correlation coefficient analysis showed that DR severity was significantly positively correlated with PlGF (r = 0.22, *p* = 0.002), VEGF-D (r = 0.25, *p* < 0.001), VEGF-C (r = 0.15, *p* = 0.048) and VEGF-A (r = 0.17, *p* = 0.031) but negatively correlated with MCP-1 (r = −0.15, *p* = 0.043). IL-6 (r = −0.05, *p* = 0.483) and IL-8 (r = 0.05, *p* = 0.498) were also correlated with the development of DR, but the statistical difference was not significant (Figure 2).

### 3.6. Multivariable multinomial logistic regression analysis

**Figure 3:**
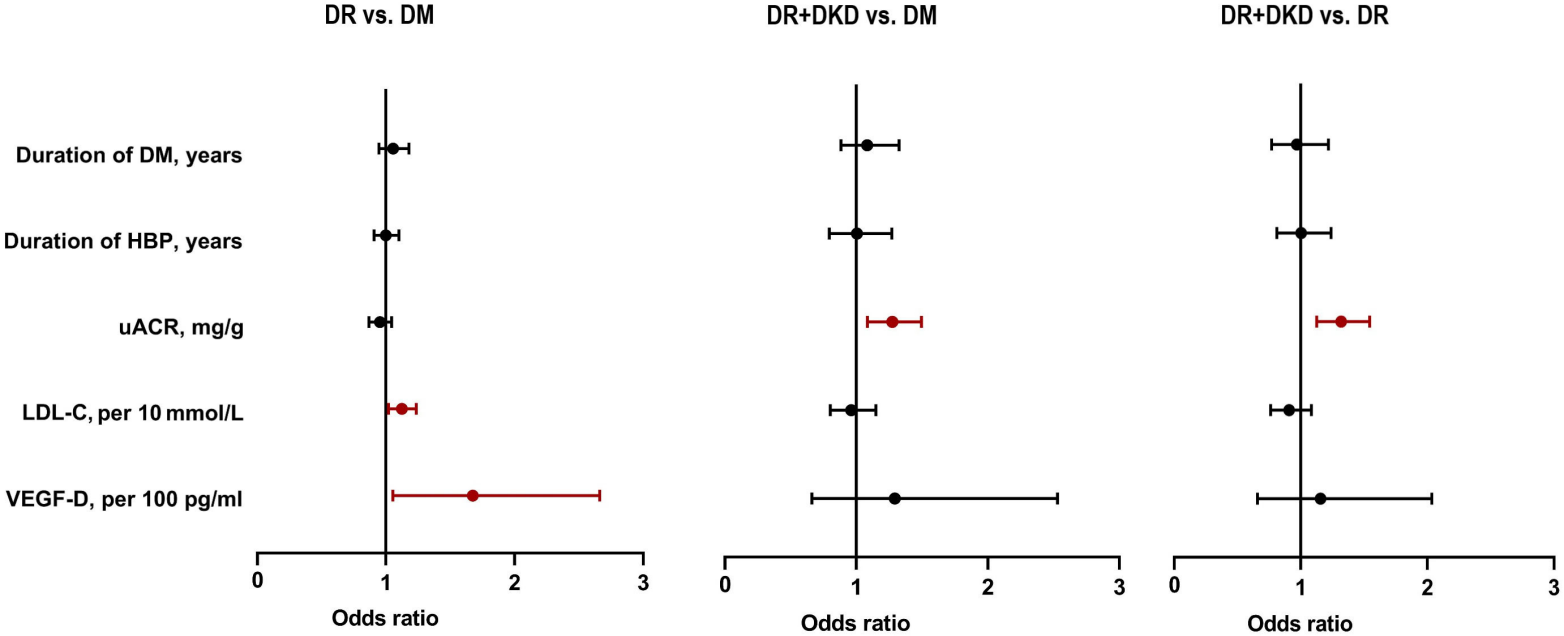
Multivariate logistic regression analysis showing uACR, LDL-C, and VEGF-D were risk factors for DR and DKD. DM, diabetes mellitus; DR, diabetic retinopathy; DKD, diabetic kidney disease; HBP, high blood pressure; LDL-C, low-density lipoprotein cholesterol; uACR, urinary albumin/creatinine ratio; VEGF, vascular endothelial growth.

By using multivariable multinomial logistic regression analysis, when DM was considered as the independent variable, LDL-C per 10 (OR = 1.122, 95%CI 1.019–1.235, *p* = 0.019) and VEGF-D per100 (OR = 1.674, 95%CI 1.053–2.661, *p* = 0.029) were significantly different between the DR and DM group (Figure 3). When DM was considered as the independent variable, uACR was significantly different in DR+DKD vs. DM group (OR = 1.273, 95%CI 1.083–1.495, *p* = 0.003) and in DR+DKD vs. DR group (OR = 1.318, 95%CI 1.125–1.544, *p* = 0.001) (Figure 3).

### 3.7. The relationship between retinal and renal microvasculopathy

**Figure 4:**
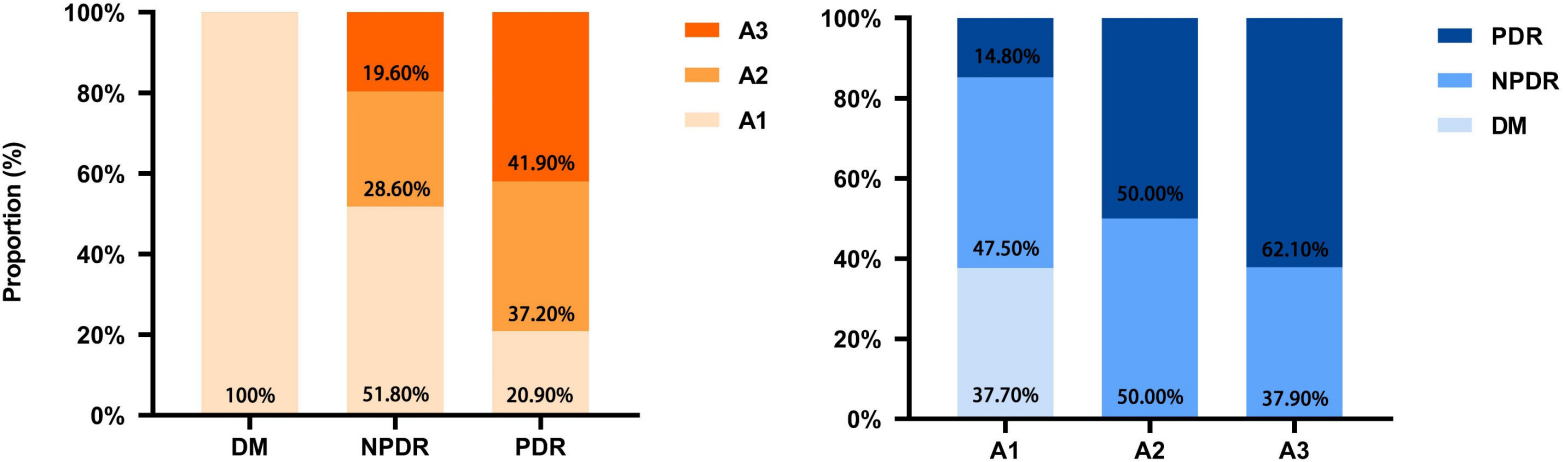
Correlation and proportion between DR and DKD grades. DM, diabetes mellitus; DR, diabetic retinopathy; DKD, diabetic kidney disease; A1, A2, A3, albuminuria categories DKD to three classes (A1–A3); A1, normal to mildly (<30 mg/g); A2, moderately (30–299 mg/g); A3, severely increased (≥300 mg/g).

Knowing that uACR (not eGFR) is closely correlated with the DR severity and a good indicator of DR progression, we further investigated the correlations between DR and DKD based on albuminuria (uACR) grades (according to the 2022 ADA grading criteria; albuminuria was graded to A1, A2, A3) (20). As shown in Figure 4, the proportion of albuminuria with A1, A2, and A3 in DM was 100%, 0% and 0%, respectively. In NPDR, the proportion of albuminuria with A1, A2, and A3 was 51.8, 28.6, and 19.6%, respectively. In PDR, the proportion of albuminuria with A1, A2, and A3 was 20.90, 37.20, and 41.90%, respectively; the difference between the three groups was statistically significant. Furthermore, in A1 albuminuria, the proportion of DM, NPDR, and PDR was 37.70, 47.50, and 14.80%, respectively; in A2 albuminuria, the proportion of DM, NPDR, and PDR was 0, 50, and 50%, respectively; in A3 albuminuria, the proportion of DM, NPDR, and PDR was 0, 37.90, and 62.10%, respectively. The difference was statistically significant. The results indicated that kidney impairment was significantly correlated with retinal microvasculopathy.

## 4. Discussion

In this study, we have shown that after controlling for the duration of DM and hypertension, LDL-C and circulating VEGF-D were independent risk factors for DR, while uACR was an independent risk factor for DR+DKD. DR severity was positively correlated with higher levels of albuminuria grades. These intriguing investigation results indicate that renal dysfunction is a strong pathological risk factor for DR and DKD, which is consistent with previous findings (22–27).

Blood retinal breakdown is a hallmark of DR, characterized by retinal endothelial dysfunction. Microalbuminuria is an early marker of generalized endothelial damage and is associated with an increased risk of DR (28). In a large cohort study, 10.7 mg/24 h of urine microalbumin (UMA) was shown to be a threshold that can predict the risk for the development of DR in T2DM, although it is in the traditionally accepted normal range (27). Won et al. showed that the presence of PDR is significantly associated with uACR (29). In their study, Cankurtaran et al. found that UMA was moderately correlated with the vessel density in the superficial retinal layer detected by the optical coherence tomography angiography, indicating that an elevated level of UMA could predict the early alterations in retinal microcirculation (28). Besides, a study demonstrated that remission of UMA is an independent protecting factor for the development of PDR and diabetic macular edema, and that aggressive treatment for DKD might help to prevent the progression of DR (30).

Numerous studies have shown that except UMA, eGFR (22, 24, 25, 31, 32), Scr (23, 29), AER (33, 34), and uACR (22, 32, 35), abnormal plasma phosphate (23) and renal biopsy parameters (22, 36–39) are also correlated with the occurrence and severity of DR and can further provide the pathological and mechanical evidence of the relationship between DR and DKD. The progressive narrowing and eventual occlusion of vascular lumina triggered by hyperglycemia lead to ischemia in both the retina and kidney (22). In the glomerulus, extensive capillary obstruction and podocyte loss have been found to induce urinary protein loss and decreased renal function (22). In the retina, ischemia could induce programmed cell death of endothelial, Muller and ganglion cells, leading to microvascular dysfunction, which further induces retinal hemorrhage, nicking, focal and generalized narrowing of arteriovenous (22). In this study, eGFR level was found to be in the normal range across the three groups, but lower in the DM group than the other two groups, leading to a statistically difference between the groups. This may be due to the relative shorter DM duration in the DM group, although we tried to match all the possible confounding factors that may produce bias. In the following logistic model analysis, we have controlled all the possible confounding variables, including the duration of DM. It is warranted to validate the current result by a well-designed cohort study in the near future. Additionally, uACR was abnormal in the three groups according to the DEIGO classification system. A statistically significant difference in the level of uACR was also found between the DM and DR+DKD groups (*p* < 0.001) and the DR and DR+DKD groups (*p* < 0.001). As eGFR was normal across the three groups, the difference between the groups did not mean the current results contradicted the hypothesis that DR and DKD are correlated. On the other hand, the results above confirmed our hypothesis that uACR is more sensitive than eGFR to predict the risk of DKD when the DM duration is not very long.

The similarity of the anatomical structure of the glomerulus and retina has been thought to be the pathological basis for DR and DKD. Microvessels are the common structural basis of DR and DKD Microvascular refers to the capillaries and microvascular network between tiny arteries and tiny veins with a lumen diameter < 100 μm. Microangiopathy mainly refers to the morphological changes and/or functional disorders of microvessels, microblood flow, and cells around microvessels at the microcirculation level under the action of various etiologies, resulting in corresponding clinical manifestations. Both pericytes and podocytes originate from mesenchymal cells and are important components of the outer barrier of microvessels. Pericytes are a class of pluripotent stem cells that have contractile, immune, hemostatic, phagocytic, and hemostatic effects, participating in vascular development. Podocytes are a class of highly differentiated cells that wrap on the outside of glomerular capillaries. Preclinical studies have suggested that an early stage of the pathogenesis of DR and DKD is characterized by pericyte and/or podocyte loss, basement membrane thickening, and microvascular leakage. The numbers of pericytes and podocytes decrease as diabetes progress (40). Loss of pericytes and podocytes leads to increased microvascular permeability and vascular leakage (41, 42). It was also found that serum level of VEGF is significantly increased in DR and DKD patients (43, 44).

We further found that FBG is associated with microalbuminuria and that well-controlled FBG is important for both DR and DKD, which is supported by Kundu's findings that impaired glycemic control is associated with significant elevation of urinary UMA levels (45). Impaired FBG was also identified as a good indicator of chronic kidney disease, albuminuria, or worsening kidney function by the SPRINT study (46). Furthermore, circulating levels of PlGF and VEGF-D were found to be significantly and positively correlated with the grades of uACR, indicating that circulating PlGF and VEGF-D can be used as biomarkers for retinal and renal endothelial dysfunction (43, 47, 48). These data are consistent with a clinical study (49), which found that serum levels of hypoxia-inducible factor-1 (HIF-1α), VEGF, insulin-like growth factors −1 (IGF-1), von Willebrand factor (vWf), and fibrinogen (Fg) were positively correlated with uACR, but negatively correlated with 25(OH)VD3 and eGFR, further confirming that serum HIF-1α, VEGF, vWf, and IGF-1 are involved in DKD process through endothelial injury induced by inflammation, and angiogenesis under hyperglycemia. Circulating level of PlGF was also correlated with renal microvascular dysfunction (47), albuminuria, proteinuria in patients with DKD, and retinal microvascular dysfunction in patients with DR (43). The pathological changes of the glomerular endothelial cell surface layer, including glycocalyx, is a major cause of UMA. Serum or plasma level VEGF-D has been implicated in both the blood-retinal barrier and the glomerular filtration barrier breakdown, which are the early sign of DR and DKD (43, 50). In this study, angiogenic cytokines VEGF-D and PlGF were strong risk factors for DR severity which was consistent with our previous study (43), although the participants of the current study were different from our previous study population.

In this study, highest level of LDL-C and API was found in the DR group, lower level was shown in the DR+DKD group; but the levels were quite close and did not show statistically significance [LDL-C (H = 0.745, *p* = 0.873) and API (H = 0.635, *p*= 0.431)] between the two groups. The underlying mechanism may be that LDL-C and API have been found to contribute to the occurrence and severity of DR in several clinical studies (17, 43, 51, 52), but the correlations between LDL-C and API with DKD were not supported by trials (53, 54). This result further confirmed that LDL-C and API mainly contribute to the risk of DR, not DKD. A cohort study with a large sample size is warranted to validate the current results.

Chronic inflammation and oxidative stress have been implicated in the pathogenesis in both DR and DKD. Hyperglycemia and hypertension are the most common inducers of oxidative stress and inflammation (22, 55), which contribute to the occurrence and progression of DKD and DR. In this study, we showed angiogenetic cytokines, including PlGF, VEGF-A, VEGF-C, and VEGF-D were associated with both DR and DKD. Moreover, a recent study demonstrated that low doses of erythropoietin, which is mainly produced by the kidney, could inhibit oxidative stress and early vascular changes in the experimental diabetic retina (56). In this study, we did not find the correlations between inflammatory cytokines and DR and DKD due to the limited number of enrolled subjects.

Nayak et al. showed that the increased serum sialic acid and microalbumin were strongly related to DR and DKD (57). DR and DKD could be predictors for both by Kaplan-Meier and cox proportional hazards regression model (21, 32, 58, 59). However, the onset of DKD and DR remains unknown. Studies showed that DR precedes DKD in patients with type 1 DM (24, 28), but renal injury precedes retinal damage in patients with T2DM (24). It is warranted to further investigate the underlying mechanisms of the onset of DKD and DR in patients with T2DM in a large cohort study.

In this study, we did not consider those T2DM with normal fundus but with abnormal estimated glomerular filtration (eGFR) or Albumin-to-creatinine ratio (uACR), that is DKD patients without retinopathy. According to the guideline, retinopathy is one of the important diagnostic criteria for DKD, this phenotype in clinical practice occupied very small numbers. Furthermore, these patients cannot be excluded other primary causes of kidney damage unless confirmed by a kidney biopsy, which was not accepted by the patients.

This study has some limitations. This was a case-control study, which could not provide the causative effects of the angiogenetic cytokines on DR and DKD. Also, this study has a relative sample size, and some variables did not show a significant association between DR and DKD+DR. A well-designed large cohort study is warranted to further investigate the mechanisms of the associations between DKD and DR. Furthermore, the signal transduction pathway of VEGF-D, VEGF-A, and PlGF and their regulatory effects on lipid metabolism need to be further explored.

In summary, the novelty of this study we showed that the occurrence and severity of retinal microvasculopathy were closely correlated with kidney dysfunction. Among the three kidney functional parameters, uACR resulted as the better indicator of DR severity and progression than eGFR and Scr. Also, impaired FBG was associated with microalbuminuria, emphasizing that well-controlled FBG is important for both DR and DKD. Finally, we concluded that the link between diabetic retinal and renal microvasculopathy is associated with dyslipidemia and upregulated circulating level of angiogenic cytokines.

## Data availability statement

The raw data supporting the conclusions of this article will be made available by the authors, without undue reservation.

## Ethics statement

The studies involving human participants were reviewed and approved by the Ethics Committee of Beijing Tongren Hospital, Capital Medical University. The patients/participants provided their written informed consent to participate in this study.

## Author contributions

XC: subjects enrollment, statistical analysis, and draft manuscript. XinZ: conceptualization, methodology, funding acquisition, statistical analysis, writing, and editing. ZG: statistical analysis. YY, XiaZ, QW, YW, and RX: subjects enrollment. All authors contributed to the article and approved the submitted version.

## References

[1] *IDF Diabetes Atlas 6th edition 2013*. (2013). Available online at: https://diabetesatlas.org/atlas/sixth-edition/ (acessed November 14, 2013).

[2] Zhang X Lai D Bao S Hambly BD Gillies MC. Triamcinolone acetonide inhibits p38MAPK activation and neuronal apoptosis in early diabetic retinopathy. Curr Mol Med. (2013) 13:946–58. 10.2174/1566524011313060007 23745583

[3] Yau JW Rogers SL Kawasaki R Lamoureux EL Kowalski JW Bek T. Global prevalence and major risk factors of diabetic retinopathy. Diabetes Care. (2012) 35:556–64. 10.2337/dc11-1909 22301125 PMC3322721

[4] Cruickshanks KJ Ritter LL Klein R Moss SE. The association of microalbuminuria with diabetic retinopathy. The Wisconsin Epidemiologic Study of Diabetic Retinopathy. Ophthalmology. (1993) 100:862–7. 10.1016/S0161-6420(93)31562-9 8510898

[5] Fu H Liu S Bastacky SI Wang X Tian XJ Zhou D. Diabetic kidney diseases revisited: a new perspective for a new era. Mol Metabol. (2019) 30:250–63. 10.1016/j.molmet.2019.10.005 31767176 PMC6838932

[6] Afkarian M Zelnick LR Hall YN Heagerty PJ Tuttle K Weiss NS. Clinical manifestations of kidney disease among US adults with diabetes, 1988-2014. JAMA. (2016) 316:602–10. 10.1001/jama.2016.10924 27532915 PMC5444809

[7] Zhang L Wang F Wang L Wang W Liu B Liu J. Prevalence of chronic kidney disease in China: a cross-sectional survey. Lancet. (2012) 379:815–22. 10.1016/S0140-6736(12)60033-6 22386035

[8] Cho NH Shaw JE Karuranga S Huang Y da Rocha Fernandes JD Ohlrogge AW. IDF diabetes atlas: global estimates of diabetes prevalence for 2017 and projections for 2045. Diabetes Res Clin Pract. (2018) 138:271–81. 10.1016/j.diabres.2018.02.023 29496507

[9] Group UPDSU. Effect of intensive blood-glucose control with metformin on complications in overweight patients with type 2 diabetes (UKPDS 34). Lancet. (1998) 352:854–65. 10.1016/S0140-6736(98)07037-8 9742977

[10] van Steen SC Woodward M Chalmers J Li Q Marre M Cooper ME. Haemoglobin glycation index and risk for diabetes-related complications in the action in diabetes and vascular disease: preterax and diamicron modified release controlled evaluation (ADVANCE) trial. Diabetologia. (2018) 61:780–9. 10.1007/s00125-017-4539-1 29308539 PMC6448976

[11] Group AtCCRiDF-OAESGatAtCCRiDF-OAS. Persistent effects of intensive glycemic control on retinopathy in type 2 diabetes in the action to control cardiovascular risk in diabetes (ACCORD) follow-on study. Diabetes Care. (2016) 39:1089–100. 10.2337/dc16-0024 27289122 PMC4915557

[12] Agrawal L Azad N Bahn GD Ge L Reaven PD Hayward RA. Long-term follow-up of intensive glycaemic control on renal outcomes in the Veterans Affairs Diabetes Trial (VADT). Diabetologia. (2018) 61:295–9. 10.1007/s00125-017-4473-2 29101421 PMC5747983

[13] Zoungas S. ADVANCE in context: The benefits, risks and feasibility of providing intensive glycaemic control based on gliclazide modified release. Diabetes Obes Metab. (2020) 22 (Suppl 2):5–11. 10.1111/dom.13968 32250522

[14] Xu Y Wang L Y He J Bi Li M Wang T. Prevalence and control of diabetes in Chinese adults. JAMA. (2013) 310:948–59. 10.1001/jama.2013.168118 24002281

[15] Guo K Zhang L Zhao F Lu J Pan P Yu H. Prevalence of chronic kidney disease and associated factors in Chinese individuals with type 2 diabetes: Cross-sectional study. J Diabetes Complicat. (2016) 30:803–10. 10.1016/j.jdiacomp.2016.03.020 27068269

[16] Yip W Sabanayagam C Teo BW Tay WT Ikram MK Tai ES. Retinal microvascular abnormalities and risk of renal failure in Asian populations. PLoS ONE. (2015) 10:e0118076. 10.1371/journal.pone.0118076 25658337 PMC4320082

[17] Chen X Zhang X Nie Y Gong Z Sivaprasad S Fung AT. Circulating level of homocysteine contributes to diabetic retinopathy associated with dysregulated lipid profile and impaired kidney function in patients with type 2 diabetes mellitus. Eye. (2022). 10.1038/s41433-022-02144-w. [Epub ahead of print]. 35739242 PMC10170092

[18] Introduction: Standards of Medical Care in Diabetes-2020. Diabetes Care. (2020) 43:S1–s2. 10.2337/dc20-Sint 31862741

[19] Bressler SB Ayala AR Bressler NM Melia M Qin H Ferris FL 3rd. Persistent macular thickening after ranibizumab treatment for diabetic macular edema with vision impairment. JAMA Ophthalmol. (2016) 134:278–85. 10.1001/jamaophthalmol.2015.5346 26746868 PMC4786449

[20] Introduction: Standards of Medical Care in Diabetes-2022. Diabetes Care. (2022) 45:S1–s2. 10.2337/dc22-Sint 34964812

[21] Hung CC Lin HY Hwang DY Kuo IC Chiu YW Lim LM. Diabetic retinopathy and clinical parameters favoring the presence of diabetic nephropathy could predict renal outcome in patients with diabetic kidney disease. Sci Rep. (2017) 7:1236. 10.1038/s41598-017-01204-6 28432319 PMC5430840

[22] Zhang H Wang J Ying GS Shen L Zhang Z. Diabetic retinopathy and renal function in Chinese type 2 diabetic patients. Int Urol Nephrol. (2014) 46:1375–81. 10.1007/s11255-014-0675-4 24573395

[23] Ahmed MH Elwali ES Awadalla H Almobarak AO. The relationship between diabetic retinopathy and nephropathy in Sudanese adult with diabetes: population based study. Diabetes Metab Syndr. (2017) 11 Suppl 1:S333–S6. 10.1016/j.dsx.2017.03.011 28325541

[24] Kotlarsky P Bolotin A Dorfman K Knyazer B Lifshitz T Levy J. Link between retinopathy and nephropathy caused by complications of diabetes mellitus type 2. Int Ophthalmol. (2015) 35:59–66. 10.1007/s10792-014-0018-6 25391917

[25] Mottl AK Kwon KS Garg S Mayer-Davis EJ Klein R Kshirsagar AV. The association of retinopathy and low GFR in type 2 diabetes. Diabetes Res Clin Pract. (2012) 98:487–93. 10.1016/j.diabres.2012.09.041 23068959 PMC4861041

[26] Manaviat MR Afkhami M Shoja MR. Retinopathy and microalbuminuria in type II diabetic patients. BMC Ophthalmol. (2004) 4:9. 10.1186/1471-2415-4-9 15228626 PMC459228

[27] Chen H Zheng Z Huang Y Guo K Lu J Zhang L. A microalbuminuria threshold to predict the risk for the development of diabetic retinopathy in type 2 diabetes mellitus patients. PLoS ONE. (2012) 7:e36718. 10.1371/journal.pone.0036718 22590593 PMC3349710

[28] Cankurtaran V Inanc M Tekin K Turgut F. Retinal microcirculation in predicting diabetic nephropathy in type 2 diabetic patients without retinopathy. Ophthalmologica. (2020) 243:271–9. 10.1159/000504943 31775153

[29] Lee WJ Sobrin L Lee MJ Kang MH Seong M Cho H. The relationship between diabetic retinopathy and diabetic nephropathy in a population-based study in Korea (KNHANES V-2, 3). Invest Ophthalmol Vis Sci. (2014) 55:6547–53. 10.1167/iovs.14-15001 25205863

[30] Hsieh YT Hsieh MC. Time-sequential correlations between diabetic kidney disease and diabetic retinopathy in type 2 diabetes - an 8-year prospective cohort study. Acta Ophthalmol. (2021) 99:e1–6. 10.1111/aos.14487 32567151

[31] Zhang X Kumari N Low S Ang K Yeo D Yeoh LY. The association of serum creatinine and estimated glomerular filtration rate variability with diabetic retinopathy in Asians with type 2 diabetes: a nested case-control study. Diab Vasc Dis Res. (2018) 15:548–58. 10.1177/1479164118786969 30014713

[32] Rodriguez-Poncelas A Mundet-Tuduri X Miravet-Jimenez S Casellas A. Barrot-De la Puente JF Franch-Nadal J. Chronic kidney disease and diabetic retinopathy in patients with type 2 diabetes. PLoS ONE. (2016) 11:e0149448. 10.1371/journal.pone.0149448 26886129 PMC4757564

[33] Kramer CK Retnakaran R. Concordance of retinopathy and nephropathy over time in Type 1 diabetes: an analysis of data from the Diabetes Control and Complications Trial. Diabet Med. (2013) 30:1333–41. 10.1111/dme.12296 23909911

[34] Stephenson JM Fuller JH Viberti GC Sjolie AK Navalesi R. Blood pressure, retinopathy and urinary albumin excretion in IDDM: the EURODIAB IDDM complications study. Diabetologia. (1995) 38:599–603. 10.1007/BF00400730 7489844

[35] Pang C Jia L Jiang S Liu W Hou X Zuo Y. Determination of diabetic retinopathy prevalence and associated risk factors in Chinese diabetic and pre-diabetic subjects: Shanghai diabetic complications study. Diabetes Metab Res Rev. (2012) 28:276–83. 10.1002/dmrr.1307 22139892

[36] Klein R Zinman B Gardiner R Suissa S Donnelly SM Sinaiko AR. The relationship of diabetic retinopathy to preclinical diabetic glomerulopathy lesions in type 1 diabetic patients: the renin-angiotensin system study. Diabetes. (2005) 54:527–33. 10.2337/diabetes.54.2.527 15677511

[37] Zhang J Wang Y Li L Zhang R Guo R Li H. Diabetic retinopathy may predict the renal outcomes of patients with diabetic nephropathy. Ren Fail. (2018) 40:243–51. 10.1080/0886022X.2018.1456453 29633887 PMC6014304

[38] Moriya T Matsubara M Kishihara E Yoshida Y Ouchi M. Type 2 diabetic patients with diabetic retinopathy and concomitant microalbuminuria showed typical diabetic glomerulosclerosis and progressive renal dysfunction. J Diabetes Complicat. (2016) 30:1111–6. 10.1016/j.jdiacomp.2016.04.007 27138869

[39] Yamanouchi M Mori M Hoshino J Kinowaki K Fujii T Ohashi K. Retinopathy progression and the risk of end-stage kidney disease: results from a longitudinal Japanese cohort of 232 patients with type 2 diabetes and biopsy-proven diabetic kidney disease. BMJ Open Diabetes Res Care. (2019) 7:e000726. 10.1136/bmjdrc-2019-000726 31798893 PMC6861100

[40] Hammes HP Lin J Wagner P Feng Y Vom Hagen F Krzizok T. Angiopoietin-2 causes pericyte dropout in the normal retina: evidence for involvement in diabetic retinopathy. Diabetes. (2004) 53:1104–10. 10.2337/diabetes.53.4.1104 15047628

[41] Ferland-McCollough D Slater S Richard J Reni C Mangialardi G. Pericytes, an overlooked player in vascular pathobiology. Pharmacol Ther. (2017) 171:30–42. 10.1016/j.pharmthera.2016.11.008 27916653 PMC6008604

[42] Laredo F Plebanski J Tedeschi A. Pericytes: problems and promises for CNS repair. Front Cell Neurosci. (2019) 13:546. 10.3389/fncel.2019.00546 31866833 PMC6908836

[43] Zhang X Qiu B Wang Q Sivaprasad S Wang Y Zhao L. Dysregulated serum lipid metabolism promotes the occurrence and development of diabetic retinopathy associated with upregulated circulating levels of VEGF-A, VEGF-D, and PlGF. Front Med. (2021) 8:779413. 10.3389/fmed.2021.779413 34904074 PMC8664628

[44] Wei Y Han S Zhou R Xu P Zhou L Zhu Z. Increased serum VEGF-B level is associated with renal function impairment in patients with type 2 diabetes. Front Endocrinol. (2022) 13:862545. 10.3389/fendo.2022.862545 35399943 PMC8988280

[45] Kundu D Roy A Mandal T Bandyopadhyay U Ghosh E Ray D. Relation of microalbuminuria to glycosylated hemoglobin and duration of type 2 diabetes. Niger J Clin Pract. (2013) 16:216–20. 10.4103/1119-3077.110159 23563465

[46] Drawz PE Pajewski NM Bates JT Bello NA Cushman WC Dwyer JP. Effect of intensive versus standard clinic-based hypertension management on ambulatory blood pressure: results from the SPRINT (Systolic Blood Pressure Intervention Trial) Ambulatory Blood Pressure Study. Hypertension. (2017) 69:42–50. 10.1161/HYPERTENSIONAHA.116.08076 27849563 PMC5145774

[47] Li J Cao Y Liu W Wang Q Qian Y Lu P. Correlations among diabetic microvascular complications: a systematic review and meta-analysis. Sci Rep. (2019) 9:3137. 10.1038/s41598-019-40049-z 30816322 PMC6395813

[48] Williams MD Nadler JL. Inflammatory mechanisms of diabetic complications. Curr Diab Rep. (2007) 7:242–8. 10.1007/s11892-007-0038-y 17547842

[49] Shao Y Lv C Yuan Q Wang Q. Levels of Serum 25(OH)VD3, HIF-1α, VEGF, vWf, and IGF-1 and their correlation in type 2 diabetes patients with different urine albumin creatinine ratio. J Diabetes Res. (2016) 2016:1925424. 10.1155/2016/1925424 27069929 PMC4812448

[50] Nguyen TTU Kim H Chae YJ Jung JH Kim W. Serum VEGF-D level is correlated with renal dysfunction and proteinuria in patients with diabetic chronic kidney disease. Medicine (Baltimore). (2022) 101:e28804. 10.1097/MD.0000000000028804 35363168 PMC9282107

[51] Zhang X Nie Y Gong Z Zhu M Qiu B Wang Q. Plasma apolipoproteins predicting the occurrence and severity of diabetic retinopathy in patients with type 2 diabetes mellitus. Front Endocrinol. (2022) 13:915575. 10.3389/fendo.2022.915575 35937834 PMC9353260

[52] Rahman MH Kamrul-Hasan AB Islam MR Hasan AY Chowdhury FQ Miah OF. Frequency and risk factors of diabetic retinopathy among patients with type 2 diabetes mellitus: a single-center study from Bangladesh. Mymensingh Med J. (2020) 29:807–14. 33116081

[53] Tu ST Chang SJ Chen JF Tien KJ Hsiao JY Chen HC. Prevention of diabetic nephropathy by tight target control in an asian population with type 2 diabetes mellitus: a 4-year prospective analysis. Arch Intern Med. (2010) 170:155–61. 10.1001/archinternmed.2009.471 20101010

[54] Gong L Wang C Ning G Wang W Chen G Wan Q. High concentrations of triglycerides are associated with diabetic kidney disease in new-onset type 2 diabetes in China: Findings from the China Cardiometabolic Disease and Cancer Cohort (4C) Study. Diabetes Obes Metab. (2021) 23:2551–60. 10.1111/dom.14502 34322974 PMC9291490

[55] Lopes de Faria JB Silva KC Lopes de Faria JM. The contribution of hypertension to diabetic nephropathy and retinopathy: the role of inflammation and oxidative stress. Hypertens Res. (2011) 34:413–22. 10.1038/hr.2010.263 21228783

[56] Wang Q Pfister F Dorn-Beineke A. vom Hagen F Lin J Feng Y. Low-dose erythropoietin inhibits oxidative stress and early vascular changes in the experimental diabetic retina. Diabetologia. (2010) 53:1227–38. 10.1007/s00125-010-1727-7 20339831

[57] Nayak BS Roberts L. Relationship between inflammatory markers, metabolic and anthropometric variables in the Caribbean type 2 diabetic patients with and without microvascular complications. J Inflamm. (2006) 3:17. 10.1186/1476-9255-3-17 17187674 PMC1764741

[58] Butt A Mustafa N Fawwad A Askari S Haque MS Tahir B. Relationship between diabetic retinopathy and diabetic nephropathy; a longitudinal follow-up study from a tertiary care unit of Karachi, Pakistan. Diabetes Metab Syndr. (2020) 14:1659–63. 10.1016/j.dsx.2020.08.026 32898743

[59] Jeng CJ Hsieh YT Yang CM Yang CH Lin CL Wang IJ. Diabetic retinopathy in patients with diabetic nephropathy: development and progression. PLoS ONE. (2016) 11:e0161897. 10.1371/journal.pone.0161897 27564383 PMC5001700

